# Efficacy and Safety of Fosfomycin Disodium in Patients with Bacterial Infections: A Single-Center, Real-Life Clinical Study

**DOI:** 10.3390/jcm14124386

**Published:** 2025-06-19

**Authors:** Fabio Luciano, Lorenzo Bertolino, Fabian Patauner, Filomena Boccia, Raffaella Gallo, Pino Sommese, Anna Maria Carolina Peluso, Oriana Infante, Silvia Mercadante, Augusto Delle Femine, Arta Karruli, Roberto Andini, Rosa Zampino, Emanuele Durante-Mangoni

**Affiliations:** 1 Department of Advanced Medical and Surgical Sciences and Department of Precision Medicine, University of Campania ‘L. Vanvitelli’, Via De Crecchio 7, 80138 Naples, Italy; fabio.luciano@studenti.unicampania.it (F.L.); lorenzo.bertolino@unicampania.it (L.B.); fabian.patauner@unicampania.it (F.P.); raffaella.gallo@unicampania.it (R.G.); sommesepino@gmail.com (P.S.); annamariapeluso24@gmail.com (A.M.C.P.); infanteoriana32@gmail.com (O.I.); silvia.mercadante@virgilio.it (S.M.); augusto.dellefemine1@gmail.com (A.D.F.); arta.karruli@gmail.com (A.K.); rosa.zampino@unicampania.it (R.Z.); 2 Unit of Internal Medicine and Transplants, AORN Ospedali dei Colli-Monaldi Hospital, Piazzale E. Ruggieri, 80131 Naples, Italy; filomena.boccia@unicampania.it (F.B.); roberto.andini@ospedalideicolli.it (R.A.)

**Keywords:** fosfomycin, safety, infectious disease, antibiotics, efficacy

## Abstract

**Objectives:** Fosfomycin is an old antibiotic that has recently gained attention owing to its preserved activity against multidrug-resistant (MDR) bacteria. Data on its use in real life are limited. Thus, we evaluated the efficacy and safety of fosfomycin disodium in the context of our hospital clinical practice. **Methods:** Single-center, retrospective, observational study on 56 patients who received fosfomycin disodium from September 2016 to July 2023, focusing on clinical and microbiological outcomes and adverse events. **Results:** Included in this study were 56 patients. Fosfomycin disodium was administered for a median duration of 10 days [5–13.5] and was always used in combination with other antibiotics, more frequently with meropenem (16 cases, 28.6%) and colistin (11 cases, 19.6%). It was mostly used for treating pneumonia (41%), followed by bloodstream infections (19.6%), urinary tract infections (16.1%), bone infections (16.1%), and surgical site infections (7.1%). The most common isolated pathogen was Pseudomonas aeruginosa (17%), and polymicrobial infections were detected in 18 patients (32%). Among the isolated bacteria, 36 (44.4%) were MDR. The complete resolution, defined as the disappearance of symptoms, eradication of the causative microorganism, and decrease in CRP levels, was achieved in 39% of cases. During treatment, we observed electrolyte imbalances, in particular a decrease in serum potassium (0.6 mEq/L [0.3–1.1]), calcium (0.7 mEq/L [0.3–1.1]) and magnesium levels (0.3 mg/dL [0.20–0.48]), and an increase in serum sodium levels (4 mEq/dL [2–7]). Changes in potassium and sodium levels were more pronounced in patients with prior kidney dysfunction and heart failure, respectively, and in patients receiving fosfomycin diluted with saline compared with 5% glucose solution (*p* = 0.04). **Conclusions:** Fosfomycin is effective in treating complicated infections in comorbid patients when combined with other antimicrobials. During treatment, major electrolyte imbalances occur that require careful monitoring and correction, especially in patients with prior kidney disease.

## 1. Introduction

The spread of multidrug-resistant (MDR) and extensively drug-resistant (XDR) bacteria is currently raising concern and interest worldwide due to growing mortality rates [1]. Newer antibiotic options to treat such infections are increasingly available [2], but tackling MDR/XDR pathogens remains challenging in frail, comorbid patients. These infections also have a substantial impact on healthcare resource utilization [3].

Consequently, clinicians are forced to still consider the use of older molecules that might have maintained activity against MDR/XDR pathogens while preserving good tolerability and favorable cost-effectiveness. Fosfomycin, an antibiotic discovered in 1969 that has recently been made available as an i.v. formulation for severe infections, is one of these old-revived antibiotics [4]. Indeed, it has broad-spectrum activity with bactericidal effects against various pathogens, including methicillin-resistant *Staphylococcus aureus* (*MRSA*), glycopeptide-resistant *enterococci,* and multidrug-resistant *Enterobacterales* [5]. For these reasons, fosfomycin could be considered in the context of combination regimens for the treatment of polymicrobial infections or in the empirical therapy of infections in seriously ill patients when early appropriate (covering) therapy is vital [6,7]. Indeed, intravenous fosfomycin disodium is currently licensed for treating patients with complicated urinary tract infections, infective endocarditis, bone and joint infections, hospital-acquired pneumonia (including ventilator-associated pneumonia), complicated skin and soft-tissue infections, bacterial meningitis, complicated intra-abdominal infections, and bacteremia that occur in association with or are suspected to be associated with any of the above-mentioned infections [8]. Owing to its very low molecular weight [9], fosfomycin presents extensive tissue penetration that includes the central nervous system (CNS), bones, joints, and soft tissues, which are usually the most difficult compartments to penetrate [10]. Nevertheless, most of the available real-life clinical data refers to old studies, although real-life clinical data are emerging [11]. Moreover, in light of the significant sodium load associated with fosfomycin disodium administration, it seems highly clinically relevant to analyze in detail its safety in a real-life context of polymorbid, seriously ill patients.

On these bases, we conducted the present study with the aim of describing patterns of use, efficacy, and safety of fosfomycin disodium in a single academic center, in the context of real-life hospital practice.

## 2. Patients and Methods

### 2.1. Study Design and Patients Included

This was a single-center, observational, retrospective cohort study that collected data regarding the use of fosfomycin disodium at Monaldi Hospital, AORN Ospedali dei Colli, Naples, Italy, between September 2016 and June 2023. Data were retrieved from patient’s medical records stored in the digital archive of Monaldi Hospital. All patients who received at least three doses of intravenous fosfomycin disodium within this period, either as monotherapy or in combination with other antimicrobials, were included in the study. Patients who received fosfomycin disodium before being transferred to Monaldi Hospital were excluded. The study was conducted in accordance with the ethical principles of the Declaration of Helsinki and was approved by our Institutional Review Board (protocol n. 1228, 19 October 2016). Patient consent was waived due to the retrospective nature of the study.

### 2.2. Variables Analyzed

The following data were collected at baseline: demographic data (age, sex), clinical data (BMI, risk factors, and comorbidities, measured with the Charlson Comorbidity Index—CCI), features of the infective syndromes (symptoms, presence of fever, SOFA score, microbiological findings), biochemical data (blood count, renal panel, hepatic panel, electrolytes, arterial blood gases, C-reactive protein (CRP), glycemia, HS troponin, blood clotting tests, lipid profile). During hospitalization, we collected pharmacological data (administered doses of fosfomycin and other antibiotics and duration of therapy) and outcome data (duration of hospitalization, status at discharge, and infectious syndrome outcome). Clinical and infection-related data were collected before and after fosfomycin administration. We divided infective syndromes into bloodstream infections (including bacteremia and cardiovascular infections), respiratory tract infections, urinary tract infections (including pyelonephritis, complicated cystitis, and prostatitis), bone infections (including osteomyelitis and spondylodiscitis), surgical site infections (including infected surgical wounds and LVAD driveline exit-site infections). With regard to isolated pathogens, MDR was defined as bacteria resistant to at least one antimicrobial agent in three or more antimicrobial classes, while XDR was defined as nonsusceptible to at least one antimicrobial agent in all but two or fewer antimicrobial classes, according to the definitions of Magiorakos et al. [12]. Additionally, to better understand the relationships between fosfomycin administration and electrolyte imbalances, already partially described in prior literature [11,13], data regarding serum electrolyte levels were also collected and analyzed, comparing levels at the beginning and at the end of fosfomycin administration. To account for the bias produced by pharmacological correction of electrolyte imbalances, early (first 2 days) dynamics of serum electrolytes during fosfomycin treatment were also recorded.

### 2.3. Clinical and Microbiological Outcomes

Resolution of infection syndromes following administration of fosfomycin was defined on the basis of clinical improvement (fever resolution, acute physiology restoration), biochemical parameter amelioration, and microbiological eradication. Biochemical resolution was defined as any improvement of serum CRP levels; microbiological eradication was defined as clearance of the causative microorganism from the infectious source after fosfomycin treatment. According to infection status before and after administration of fosfomycin, patients were categorized into 3 groups: patients who had a *complete resolution*, with disappearance of symptoms, eradication of the causative microorganism, and decrease in CRP levels; patients who had a *partial resolution* of the infection, with microbiological eradication but no decrease in CRP levels or with decrease in CRP levels but no microbiological eradication and a variable clinical evolution; patients who had *no resolution* of the infection, who had neither microbial eradication from the initial source nor decrease in CRP levels, as well as persistence of clinical symptoms.

Adverse fosfomycin effects were defined as occurrence of any pathological change in clinical (e.g., worsening general conditions), biochemical (e.g., electrolyte imbalances, kidney or liver function test alterations), and hematological (e.g., leukopenia, thrombocytopenia) findings.

### 2.4. Statistical Analysis

Numerical variables were expressed as median and interquartile range (IQR) and were compared using the Mann–Whitney U-test (two group differences), while categorical variables were expressed as number and percentage and were compared using the chi-square test or the Fisher exact test according to numerosity of groups. Statistical tests performed were two-tailed, and the threshold of significance was set at 5% (*p* < 0.05). All analyses were carried out with the aid of SPSS 25 (IBM, Armonk, New York, NY, USA).

## 3. Results

### 3.1. Clinical Characteristics of Patients Studied

Fifty-six patients treated with fosfomycin disodium were included in this study; their main baseline characteristics are described in Table 1. Most patients were male, with a median age of 62.4 years (IQR = 55–75.3). Patients treated had a high burden of comorbidities, with a median Charlson Comorbidity Index of 4 [3,4,5,6].

The most common comorbidities included heart disease (34%), hypertension (34%), kidney disease (34%), and diabetes mellitus (34%). Twenty-seven (48%) patients were admitted to an Internal Medicine Unit, 17 (30%) to the Intensive Care Unit, 6 (11%) to the Cardio-Thoracic Surgery Unit, 4 (7%) to the Respiratory Disease Unit, and 2 (4%) to the Cardiovascular Disease Unit.

### 3.2. Clinical Syndromes and Microbial Etiology

Fosfomycin disodium was mostly used to treat respiratory tract infections (23 cases, 41.1%), followed by bloodstream infections (11 patients, 19.6%), urinary tract infections (9 patients, 16.1%), bone infections (9 patients, 16.1%), and surgical site infections (4 patients, 7.1%).

**Figure 1:**
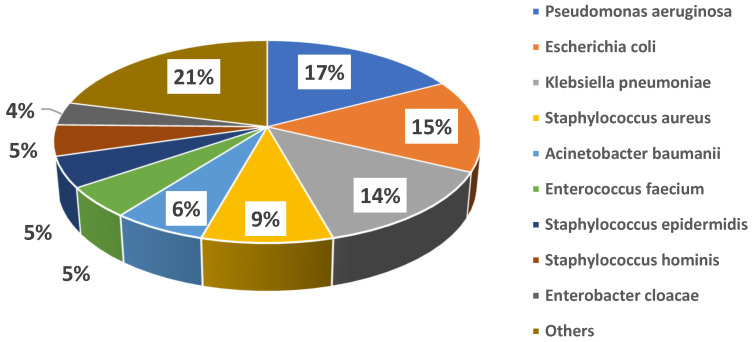
Distribution of major causative bacteria in the study cohort.

Microbiological cultures were performed at baseline in all cases, with a total of 72 microbiology samples, 3 (4%) of which were negative and 69 (96%) positive, with isolation of 81 pathogens. In detail, the following cultures were obtained: 28 (39%) blood cultures, 20 (28%) cultures from respiratory samples, 7 (10%) urine cultures, 11 (15%) swabs from different sites (8, 73% of which, were from surgical sites; 2, 18%, from the exit site of left ventricle assist device drivelines; 1, 9%, from an entero-cutaneous fistula), 3 (4%) cultures from synovial fluid, 1 (1%) culture from abscess drainage, and 1 (1%) culture from a surgically removed valve; in only one case was fosfomycin treatment initiated targeting a pathogen cultured from a rectal swab, due to lack of any other findings in a patient with sepsis. Figure 1 shows the details of isolated bacteria. The most common pathogens isolated were *Pseudomonas aeruginosa* (14 cases, 17%), *Escherichia coli* (12 cases, 15%), *Klebsiella pneumoniae* (11 cases, 14%), coagulase-negative *Staphylococci* (11 cases, 14%), and *Staphylococcus aureus* (7 cases, 9%). Overall, 18 (32%) patients showed polymicrobial infections. MDR strains were isolated among both Gram-negative (21/53, 40%) and Gram-positive (15/28, 54%) bacteria. Individual species distribution is shown in the Supplementary Table S1, whereas the major antimicrobial resistance patterns are reported in the Supplementary Table S2.

The 81 pathogens isolated were responsible for 38 (68%) mono-microbial infections and 18 (32%) poly-microbial infections; in the polymicrobial infection group, all pathogens were isolated from the same source in 4 (22%) cases and from different sources in 14 (78%) cases.

The distribution of pathogens in the context of the different clinical syndromes is detailed in Supplementary Table S3.

After the administration of fosfomycin, follow-up cultures became negative in 25 cases (45%), while 10 cultures (18%) remained positive for the same pathogen. In 20 cases (36%), follow-up cultures were not performed. Specifically, there were 20 (36%) patients with positive blood cultures at the start of fosfomycin treatment; of them, 11 (55%) cleared blood cultures during fosfomycin-based treatment.

Overall, sensitivity to fosfomycin was tested on 25 pathogens before antimicrobial treatment, and 6 of them (24%) turned out to be resistant to fosfomycin; nonetheless, fosfomycin was maintained in these cases in combination treatment schemes due to a lack of effective alternatives, poor clinical conditions, or appropriate infective syndrome. Among bacteria resistant to fosfomycin in vitro, 2 *Klebsiella pneumoniae* isolated from sputum and blood culture and 1 case of *Staphylococcus aureus* from sputum persisted in follow-up cultures. Other bacteria (including strains of *Klebsiella pneumoniae*, *Pseudomonas aeruginosa*, *MR-Staphylococcus aureus*, *Staphylococcus haemolyticus*, *Enterobacter aerogenes*, *Enterobacter cloacae*, *Enterococcus faecium*, *Corynebacterium striatum*, and *Stenotrophomonas maltophilia*), which tested sensitive to fosfomycin, also persisted in follow-up cultures after treatment.

### 3.3. Patterns of Fosfomycin Use

Forty-one (73%) patients had been treated with other antibiotics before starting fosfomycin.

**Figure 2:**
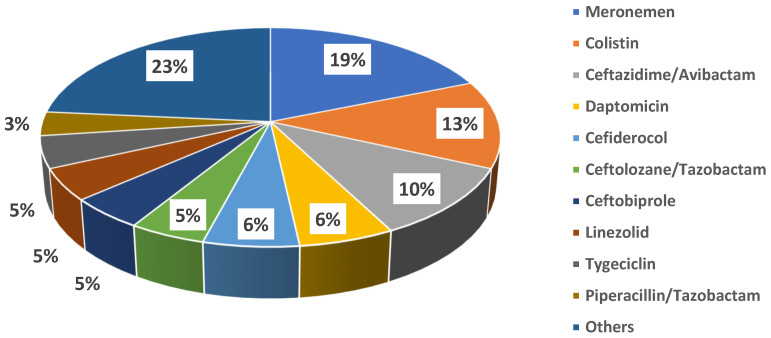
Antibiotics co-administered with fosfomycin.

Fosfomycin was administered for a median duration of 10 days [5–13.5]. Specifically, the median duration of therapy for bloodstream infection was 15 days [9–19.5], being the longest, followed by bone infections (median = 13 [6–26]), surgical site infections (median = 9.5 [7.5–10.8], respiratory tract infections (median = 8 [5–12]), and urinary tract infections (median = 7 [5–10]. The daily dose changed according to the different clinical syndromes. The fosfomycin dose was adjusted for renal function after a full dose, equal to 8 grams, was given to all patients on the first day of treatment. Fosfomycin was always used in combination with other antibiotics (100% of cases). Molecules more frequently combined were meropenem (16 cases, 28.6%) and colistin (11 cases, 19.6%); other antibiotics co-administered with fosfomycin are shown in Figure 2.

Details of fosfomycin use are shown in Table 2.

### 3.4. Clinical Outcomes

According to the predefined outcomes, patients were categorized into three groups: twenty-two (39%) patients achieved *complete resolution* of the infective syndrome, and ninetten (86%) of them were alive at the end of treatment; twenty-eight (50%) patients experienced *partial resolution*, and twenty-one (75%) of them were alive at the end of the treatment; one (2%) patient had *no resolution* and died during treatment. For five patients (9%), there were not enough data to assess their outcomes. Overall, all patients died from infectious complications, although in two cases, death was also related to complications of cardiac surgery and neurological deterioration. The outcome distribution is shown in Table 3.

Moreover, we compared primary and secondary outcomes according to Gram-positive and Gram-negative infections and infective syndromes. As we show in Supplementary Table S4, outcomes were significantly different according to infective syndrome (*p* = 0.007) but not the causative pathogen group.

### 3.5. Changes in Hematochemical Parameters

As shown in Table 4, significant changes in laboratory parameters were observed, mostly reduction in CRP levels (*p* < 0.01) and neutrophil count (*p* = 0.01). With the exclusion of serum electrolytes, no other significant changes were observed in the remaining laboratory parameters (Table 4).

#### Electrolyte Imbalances

**Figure 3:**
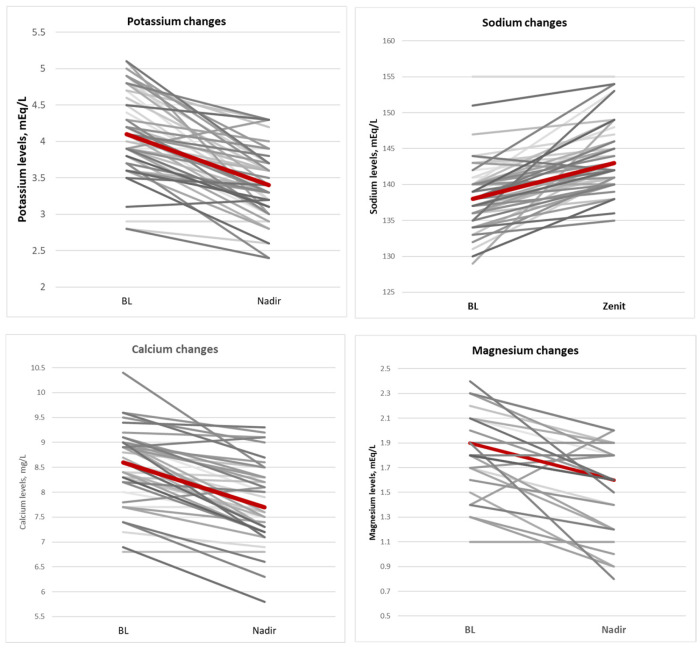
Central tendency of major electrolytes imbalance.

During fosfomycin treatment, electrolyte imbalances were often observed; specifically, a significant increase in serum sodium concentrations (median = 4 [2–7] *p* < 0.01) and a significant decrease in serum potassium (median = 0.6 [0.3–1.10] *p* < 0.01), calcium (median = 0.7 [0.3–1.10] *p* < 0.01), and magnesium (median= 0.3 [0.2–0.5] *p* = 0.01) concentrations occurred. Electrolyte changes improved after correction was carried out with potassium-sparing diuretics in 20 patients (36%), potassium chloride or potassium aspartate i.v. infusions in 34 patients (61%), calcium gluconate i.v. infusion in 22 patients (39%), and magnesium i.v. infusion in 20 patients (36%). Figure 3 shows the central tendency of major electrolyte imbalance.

Although not significant, the decrease in serum potassium level was larger in patients with prior chronic kidney disease (median decrease of 1 mEq/L) than in patients with a normal renal function at baseline (median decrease of 0.6 mEq/L; *p* = 0.18). Interestingly, renal functional parameters improved overall during fosfomycin treatment in the study cohort (Table 4), although the observed differences were not significant. Also, sodium retention was greater in patients with prior heart disease (median increase of 5.5 mEq/L) than in those with normal cardiac function (median increase of 3 mEq/L, *p* = 0.06).

Fosfomycin was dissolved, according to the manufacturer’s recommendation, with 0.9% sodium chloride solution in 11 cases (20%) or with 5% glucose solution in 33 cases (59%), while drug dilution was unknown in the remaining 12 cases. A sub-analysis comparing electrolyte changes in patients receiving fosfomycin diluted in 0.9% sodium chloride or 5% glucose showed that electrolyte correction was needed in 91% of the former compared with 57.6% of the latter patients (*p* = 0.04).

As shown in Supplementary Table S5, chronic kidney disease, but not heart failure, was significantly associated with electrolyte imbalance development during fosfomycin administration.

### 3.6. Outcomes of Hospitalization and All-Cause Mortality

Fourteen (28%) patients died during the hospitalization. Death occurred during fosfomycin treatment in only five of these patients (36%), of whom two were suffering from both end-stage heart and renal failure, one from coronary artery disease, and one from chronic kidney disease, all conditions contributing to the fatal outcome.

Table 3 shows the main differences between patients who were alive or dead at the end of fosfomycin treatment. Patients who died were more frequently dwelling in the ICU (*p* < 0.01), with lower respiratory tract infection (*p* = 0.01), and showing *E. faecium* and *S. haemolyticus* as the causative agents of the infection. In contrast, a favorable outcome was associated with admission to an Internal Medicine Unit (*p* < 0.01).

Among patients alive at the end of hospitalization, twenty-two (52%) achieved a microbiological cure, and eight (19%) had no microbiological eradication, while microbiological data were not available for the remaining 12 cases (29%).

## 4. Discussion

Being an old molecule approved several decades ago, efficacy and safety data regarding fosfomycin clinical use, generated by means of current clinical trial methodology, are lacking [5], the only exception being complicated urinary tract infections [14]. Accordingly, clinical decision-making should rely on evidence coming from field studies in approved indications. As a matter of fact, real-life clinical data on the new formulation of fosfomycin disodium are also limited as of today. Notwithstanding, fosfomycin continues to hold a valuable therapeutic role for complicated infections caused by multidrug-resistant bacteria. These pathogens often pose treatment challenges due to limited therapeutic options, but resistance to fosfomycin remains low.

Our study in the context of a tertiary care real-life practice suggests that fosfomycin is effective when used in predominantly complicated infections and in comorbid patients. Effectiveness was assessed through monitoring of infection signs, serum CRP levels, and microbiological outcomes. Our data show that in most patients, fosfomycin administration translated into a favorable clinical response, with complete resolution of infection signs, including CRP level reduction and microbiological eradication, in nearly 40% of cases and partial resolution of signs/symptoms in half of patients assessed. Clinical response correlated with positive hospitalization outcomes, suggesting that it would be rational to conduct prospective studies in the present infectious syndromes and in combination with the antibiotics used in our cohort to more accurately evaluate fosfomycin clinical effectiveness. Our findings align with the result from previous research by P. Savaj et al. [15].

Despite the renal excretion of fosfomycin and the associated high burden of sodium load, our data show its use translated into an average, although not statistically significant, reduction in serum creatinine levels and a consequent improvement in glomerular filtration rate; this suggests a potentially favorable impact on renal function, which is particularly important in severely ill patients with multiple comorbidities, whose renal function is often compromised. This observation is particularly important given the potential renal toxicity of several other antibiotics commonly used alongside fosfomycin.

In this study, fosfomycin’s impact on serum electrolyte balance was analyzed in depth. Our observations provide what we believe is highly clinically significant information. Specifically, we observed a significant increase in serum sodium levels, as already demonstrated in previous studies [11,16,17,18]. This increase, due to the high content of sodium in current the i.v. fosfomycin formulation, was independent of renal function. However, it was more pronounced in patients suffering from heart failure compared with those with normal cardiac function, suggesting additional caution should be exercised in this context rather than in patients with prior kidney disease.

Additionally, we observed a major decrease in serum potassium levels during fosfomycin administration, necessitating pharmacological correction. The reduction in potassium levels was numerically larger in patients suffering from prior chronic kidney disease and correlated with a greater decrease in serum creatinine levels. Thus, considering the renal functional improvement induced by fosfomycin, our data suggest that an increased renal excretion of potassium could underlie the hypokalemia commonly observed on fosfomycin disodium treatment.

We next analyzed the effects of diuretics, often co-administered in seriously ill patients, on electrolyte abnormalities during fosfomycin administration. Interestingly, there was no significant difference in serum potassium reduction between patients who did or did not receive treatment with potassium-sparing diuretics during fosfomycin treatment. Also, potassium levels decreased to the same extent in those who had already been receiving potassium-sparing diuretics prior to fosfomycin treatment. This data may indicate that aldosterone plays no significant role in the mechanism of potassium loss induced by fosfomycin. Instead, the high sodium concentration reaching the distal convoluted and collecting tubules during treatment with fosfomycin is likely responsible for the potassium loss [19]. Further studies are underway in our hospital to better characterize these pathophysiological changes in fosfomycin-treated subjects.

We also compared serum sodium changes in patients who received fosfomycin diluted in 0.9% saline solution versus those who received it in a 5% glucose solution. Patients treated with saline solution experienced significantly higher serum sodium levels, suggesting that 5% glucose solution may be the preferable drug diluent to mitigate sodium overload.

Drops in serum calcium and magnesium were also noted, although these did not relate to renal function variation. Most likely, the reduction in serum magnesium may be linked to the reduced sodium reabsorption [20]. Conversely, in relation to sodium excretion coupled with calcium, sodium overload induced by fosfomycin may exacerbate this physiological mechanism and provoke the calcium decrease observed [21], along with the potential effects of hypomagnesemia on impaired secretion of parathormone [20]. Nevertheless, we did not find similar literature data about these findings; thus, further research on electrolyte imbalance during fosfomycin treatment is warranted. At present, we strongly suggest closely monitoring serum electrolytes during fosfomycin administration and correcting any abnormalities according to protocols.

Of the fifty-six patients treated with fosfomycin, only five died during treatment. Notably, these patients suffered from advanced CKD and from end-stage heart failure (HF). Considering the favorable impact of fosfomycin in patients with impaired renal function, our data suggest a role of HF in affecting these outcomes due to the increased fluid overload. Thus, careful monitoring of cardiac function and fluid status is strongly recommended during the treatment of patients with heart failure, as observed in three reports [13,22,23], which also recommend considering systemic and pulmonary congestion and monitoring NT-proBNP serum levels in these patients [23].

## 5. Study Limitations

This was a retrospective, observational study, including a relatively small number of patients. As per the study design, some data from individual patients were missing. In addition, due to the retrospective nature of the study, different infectious sites, different pathogens, and different fosfomycin dosing regimens had to be included.

## 6. Conclusions

Despite the overall favorable outcomes, fosfomycin demonstrated a safety profile characterized by considerable electrolyte changes. Thus, special attention should be given to monitoring and correcting electrolyte imbalances during treatment with fosfomycin, especially in patients suffering from HF. Diluting fosfomycin in a glucose solution rather than saline is advised to reduce the extent of sodium overload.

## Figures and Tables

**Table 1:** Clinical characteristics of patients at baseline.

Number	56
Age, median [IQR]	62.4 [30–75]
Sex (M/F)—n. (%)	38 (68)/18 (32)
BMI, median [IQR]	26.7 [23.6–30]
Pack/years, median [IQR]	49.9 [30–75]
CCI, median [IQR]	4 [3–6]
Haemoglobin, g/dL, median [IQR]	9.7 [8.1–10.75]
Erytrocytes, cells/μL, median [IQR]	3460 [2850–3780]
Leukocytes, cells/μL, median [IQR]	9920 [7150–14,777]
Neutrophils, cells/μL, median [IQR]	8340 [6130–13,000]
Platelets, cells/μL, median [IQR]	210 [115–303]
INR, median [IQR]	1.2 [1.13–1.38]
aPTT, s, median [IQR]	33.5 [30.95–40.45]
C-Reactive Protein, mg/dL, median [IQR]	11.6 [5.7–18.35]
Creatinine, mg/dL, median [IQR]	1 [0.6–1.7]
Estimated GFR, mL/min, median [IQR]	78.5 [41.5–107.8]
Urea, mg/dL, median [IQR]	52 [31–96.5]
Fasting Glucose, mg/dL, median [IQR]	107 [88.25–137.5]
AST, IU/L, median [IQR]	25 [15–39]
ALT, IU/L, median [IQR]	24.5 [13.25–51.75]
Total Bilirubin, mg/dL, median [IQR]	0.6 [0.4–0.98]
Sodium, mEq/L, median [IQR]	138 [136–140]
Potassium, mEq/L, median [IQR]	3.9 [3.7–4.3]
Calcium, mEq/L, median [IQR]	8.6 [8–9]
Magnesium, mEq/L, median [IQR]	1.8 [1.6–2.1]
Chloride, mEq/L, median [IQR]	103 [101–106]

ALT: Alanine Transaminase; aPTT: activated Partial Thromboplastin Time; AST: Aspartate Transaminase; BMI: Body Mass Index; CCI: Charlson Comorbidity Index; F: female; GFR: Glomerular Filtration Rate; IQR: interquartile range; INR: International Normalized Ratio; M: male.

**Table 2:** Details among fosfomycin use.

Number of Patients	56
**Duration of treatment, median [IQR]**	10 [5–13.5]
**Bloodstream infections**	15 [9–19.5]
**Respiratory tract infections**	8 [5–12]
**Urinary tract infections**	7 [5–10]
**Bone Infections**	13 [6–26]
**Surgical site infections**	9.5 [7.5–10.8]
**Co-administered antibiotics, n (%)**	
**Meronemen**	16 (28.6)
**Colistin**	11 (19.6)
**Ceftazidime/Avibactam**	9 (16.1)
**Daptomicin**	5 (8.9)
**Cefiderocol**	5 (8.9)
**Ceftolozane/Tazobactam**	4 (7.1)
**Ceftobiprole**	4 (7.1)
**Linezolid**	4 (7.1)
**Tygeciclin**	4 (7.1)
**Piperacillin/Tazobactam**	3 (5.4)
**Amikacin**	2 (3.6)
**Gentamicin**	2 (3.6)
**Amoxicillin/Clavulanate**	2 (3.6)
**Cotrimoxazole**	2 (3.6)
**Teicoplanin**	2 (3.6)
**Vancomycin**	2 (3.6)
**Meropenem/vaborbactam**	2 (3.6)
**Ertapenem**	1 (1.8)
**Cefepime**	1 (1.8)
**Metronidazole**	1 (1.8)
**Ceftaroline**	1 (1.8)
**Ampicillin/Sulbactam**	1 (1.8)
**Cefazolina**	1 (1.8)
**Infection onset to start of treatment, days, median [IQR]**	7 [3–25]
**Therapeutic strategy, n (%)**	
**Empirical therapy**	29 (51.8)
**Targeted therapy**	27 (48.2)
Dosage^ in patients with impaired renal function*, mean (SD)	
**3 A (eGFR 59-45 ml/min/1.73 m^2^)**	16 (±4.3)
**3 B (eGFR 44-30 ml/min/1.73 m^2^)**	12 (±5.9)
**4 (eGFR 29-15 ml/min/1.73 m^2^)**	12 (±7)
**5/5D (eGFR < 15 ml/min/1.73 and/or dialysis)**	12 (±16)

^ Infusion duration: 3 h. * Stages of renal disfunction defined according to KDOQI Guidelines. eGFR: estimated Glomerular Filtration Rate; SD: standard deviation.

**Table 3:** Variables associated with status (alive or dead) at the end of fosfomycin treatment.

		ALIVE (42)	DEAD (14)	*p* Value
Age, median [IQR]		67.5 [56.8–70]	58.5 [31.5–75.8]	0.35
CCI, median [IQR]		4 [2.5–5.5]	5.5 [4.1–6.9]	0.13
Admission ward, n. (%)	*Cardiology Unit*	1 (2.4)	1 (7.1)	0.4
	***Respiratory Medicine Unit***	**1 (2.4)**	**3 (21.4)**	**0.04**
	*Cardiothoracic Surgery Unit*	5 (11.9)	1 (7.1)	0.62
	***Intensive Care Unit***	**8 (19.0)**	**9 (64.3)**	**<0.01**
	***Internal Medicine Unit***	**27 (64.3)**	**0 (0)**	**<0.01**
Infective syndrome, n. (%)	*Bloodstream Infections*	8 (19)	3 (21.4)	1
	***Respiratory Tract Infections***	**13 (31)**	**10 (71)**	**0.01**
	*Urinary Tract Infections*	9 (21)	0 (0)	0.09
	*Bone Infections*	9 (21)	0 (0)	0.09
	*Surgical Site Infections*	3 (7)	1 (7)	1
Causative pathogen, n. (%)	*Acinetobacter baumannii*	2 (4.8)	2 (14.3)	0.23
	*Corynebacterium striatum*	1 (2.4)	0 (0)	0.76
	*Enterobacter aerogenes*	2 (4.8)	0 (0)	0.97
	*Enterobacter cloacae*	3 7.1)	0 (0)	0.75
	*Enterococcus faecalis*	2 (4.8)	0 (0)	0.97
	***Enterococcus faecium***	**1 (2.4)**	**3 (21.4)**	**0.04**
	*Enterococcus raffinosus*	1 (2.4)	0 (0)	0.76
	*Escherichia coli*	11 (26.2)	1 (7.1)	0.13
	*Haemophilus influenza*	1 (2.4)	0 (0)	0.76
	*Hafnia alvei*	1 (2.4)	1 (7.1)	0.4
	*Klebsiella pneumonia*	8 (19.0)	4 (28.6)	0.45
	*Moraxella catarrhalis*	0 (0)	1 (7.1)	0.1
	*Pseudomonas aeruginosa*	9 (21.4)	4 (28.6)	0.58
	*Pseudomonas putida*	0 (0)	1 (7.1)	0.1
	*Sphingomonas paucimobilis*	1 (2.4)	0 (0)	0.76
	*Staphylococcus aureus*	6 (14.3)	2 (14.3)	1
	*Staphylococcus epidermidis*	2 (4.8)	2 (14.3)	0.23
	***Staphylococcus haemolyticus***	**0 (0)**	**2 (14.3)**	**0.02**
	*Staphylococcus hominis*	4 (9.5)	0 (0)	0.59
	*Staphylococcus simulans*	1 (2.4)	0 (0)	0.76
	*Stenotrophomonas maltophilia*	1 (2.4)	1 (7.1)	0.4
Main comorbidities, n. (%)	*Renal disease*	11 (26.2)	6 (42.8)	0.24
	*Heart disease*	9 (21.4)	6 (42.8)	0.12
	*Respiratory syndromes*	9 (21.4)	4 (28.5)	0.58
	*Diabetes mellitus*	12 (28.6)	4 (28.5)	1
Patients’ outcomes, n.(%)	*Complete resolution*	19 (45.2)	3 (21.4)	0.20
	*Partial resolution*	21 (50)	7 (50)	1
	*No resolution*	0 (0)	1 (7.1)	0.25
	*Not enough data*	2 (4.7)	3 (21.4)	-

*p*-value was generated by ANOVA test and Pearson’s chi-square test for median and frequency values, respectively. CCI: Charlson Comorbidity Index; IQR: Inter Quartile Range.

**Table 4:** Biochemical data at baseline (BL) and at end of fosfomycin treatment (EOT).

Parameter	BL	EOT	*p*-Value
Hematochemical Parameters	Median [IQR]	Median [IQR]	
CRP (mg/dL)	11.6 [5.7–18.3]	4.8 [2.7–8.7]	<0.01
SOFA score (0–48/72h)	3 [1–6]	3 [1–5]	0.43
RBC (cells ×10^3^/µL)	3460 [2850–3780]	3275 [2965–3795]	0.79
WBC (cells/µL)	9920 [7150–14,777]	7780 [5997–12,770]	0.08
**NEUT (cells/µL))**	**8340 [6130–13,000]**	**5730 [3815–9200]**	**0.01**
PLT (cells ×10^3^/µL)	210 [115–303]	233 [160–300]	0.32
HGB (g/dL)	9.7 [8.1–10.75]	9.1 [8.38–10.53]	0.90
CREA (mg/dL)	1 [0.6–1.7]	0.83 [0.6–1.2]	0.22
eGFR (mL/min/1.73m^2^)	78.5 [41.5–107.8]	92.7 [56–110]	0.31
UREA (mg/dL)	52 [31–96.5]	40.5 [28–70]	0.13
INR	1.22 [1.13–1.37]	1.25 [1.13–1.47]	0.65
aPTT (sec.)	33.5 [30.95–40.45]	34.55 [30.65–38.18]	0.84
AST (IU/L)	25 [15–39]	21 [14–35]	0.58
ALT (IU/L)	24.5 [13.25–51.75]	20.5 [12–42]	0.51
TOTAL BILIRUBIN (mg/dL)	0.6 [0.4–0.98]	0.55 [0.3–1.03]	0.54
**Na^+^ (mEq/L)**	**138 [136–140]**	**140 [139–143]**	**<0.01**
K^+^ (mEq/L)	3.9 [3.7–4.3]	3.9 [3.63–4.38]	0.76
Ca^2+^ (mEq/L)	8.6 [8–9]	8.15 [7.48–8.75]	0.10
Mg^2+^ (mEq/L)	1.8 [1.6–2.1]	1.8 [1.48–1.93]	0.61
Cl^−^ (mEq/L)	103 [101–106]	103 [101–105.75]	0.73

*p*-value was generated by ANOVA test and Pearson’s chi-square test for median and frequency values, respectively. ALT: Alanine Transaminase; aPTT: activated Partial Thromboplastin Time; AST: Aspartate Transaminase; BMI: Body Mass Index; CREA: Creatinine; CRP: C-reactive protein; F: female; eGFR: Glomerular Filtration Rate; HGB: Hemoglobin; IQR: interquartile range; INR: International Normalized Ratio; M: male; NEUT: Neutrophils; PLT: Platelets; RBC: Red Blood Cells; WBC: White Blood Cells.

## Data Availability

The original contributions presented in this study are included in the article/supplementary material. Further inquiries can be directed to the corresponding author(s).

## Supplementary Materials

The following supporting information can be downloaded at https://www.mdpi.com/article/10.3390/jcm14124386/s1, Table S1. Analytical presentation of bacterial species causing infections in fosfomycin-treated patients; Table S2. Major antimicrobial resistance patterns among isolates from patients treated with fosfomycin in this study; Table S3. Distribution of pathogens in the context of the different clinical syndromes. Table S4. Primary and secondary outcomes according to isolated pathogens and infective syndrome; Table S5. Univariate analysis of factors associated with electrolyte imbalance development during fosfomycin administration.
