# Supplementary material for: Efficacy and Safety of Fosfomycin Disodium in Patients with Bacterial Infections: A Single-Center, Real-Life Clinical Study
Source: J Clin Med. 2025 Jun 19;14(12):4386. doi: 10.3390/jcm14124386 (PMC12194547; doi:10.3390/jcm14124386)
Supplement: Supplementary file 1 [file jcm-14-04386-s001.zip › jcm-3430957-supplementary.pdf]

Supplementary material – Luciano F., et al.

**Supplementary Table S1. Analytical presentation of bacterial species causing infections in fosfomycin-treated patients**	
**Genus/species**	**N**
**Enterobacterales**	**30**
Enterobacter (Klebsiella) aerogenes	2
Enterobacter cloacae	3
Escherichia coli	12
Hafnia alvei	1
Klebsiella oxtytoca	1
Klebsiella pneumoniae	11
**Enterococci**	**7**
Enterococcus faecalis	2
Enterococcus faecium	4
Enterococcus raffinosus	1
**Non fermenters**	**23**
Acinetobacter baumannii	5
Moraxella catarrhalis	1
Pseudomonas aeruginosa	14
Pseudomonas putida	1
Stenotrophomonas maltophilia	2
**CN Staphylococci**	**11**
Staphylococcus epidermidis	4
Staphylococcus haemolyticus	2
Staphylococcus hominis	4
Staphylococcus simulans	1
**CP Staphylococci**	**7**
Staphylococcus aureus	7
**Others**	**3**
Corynebacterium striatum	1
Haemophilus influenzae	1
Sphingomonas paucimobilis	1
**Total**	**81**

CN: Coagulase Negative; CP: Coagulase Positive.

Supplementary Table S2. Major antimicrobial resistance patterns among isolates from patients treated with fosfomycin in this study

MDR pathogens (N=36)					
**Gram negative bacteria**		**21**	**Gram positive bacteria**		**15**
**ESBL**	Escherichia coli	3	**MR**	Staphylococci	14
	Klebsiella pneumoniae	2	**AmpR**	Enterococcus faecium	1
	Enterobacter cloacae	2			
	Enterobacter aerogenes	1			
**KPC**	Klebsiella pneumoniae	4			
**AmpC**	Escherichia coli	1			
**DTR**	Pseudomonas aeruginosa	4			
**XDR**	Acinetobacter baumannii	4			

AmpC: AmpC beta-lactamase; AmpR: Ampicillin Resistant; DTR: Difficult-to-Treat Resistance; ESBL: Extended-Spectrum Beta-Lactamase; KPC: Klebsiella pneumoniae Carbapenemase; MDR: Multi Drug Resistant; MR: Meticillin Resistant; XDR: Extensively Drug Resistant.

Supplementary Table S3. Distribution of pathogens in the context of the different clinical syndromes

Bloodstream infections	n	Lower respiratory tract infections	n	Urinary tract infections	n	Infective endocarditis	n	Surgical wound infections	n	Bone infections	n	Device-related infections	n
Staphylococcus aureus	2	Pseudomonas aeruginosa	9	Escherichia coli	5	Coagulase negative staphylococcus	3	Pseudomonas aeruginosa	3	Staphylococcus aureus	2	Pseudomonas aeruginosa	2
Coagulase negative staphylococcus	1	Coagulase negative staphylococcus	8	Klebsiella pneumoniae	3	Staphylococcus aureus	1	Staphylococcus aureus	1	Corynebacterium striatum	1	Klebsiella pneumoniae	1
Klebsiella pneumonia	1	Klebsiella pneumoniae	4			Enterococcus faecalis	1	Acinetobacter baumannii	1	Coagulase negative staphylococcus	1	Escherichia coli	1
		Escherichia coli	3			Enterococcus faecium	1	Hafnia alvei	1	Enterobacter cloacae	1		
		Acinetobacter baumannii	3			Pseudomonas aeruginosa	1			Enterococcus faecalis	1		
		Staphylococcus aureus	2			Sphingomonas paucimobilis	1			Enterococcus raffinosus	1		
		Enterobacter aerogenes	1							Escherichia coli	1		
		Enterococcus faecium	1										
		Haemophilus influenzae	1										
		Moraxella catharralis	1										

Supplementary Table S4. Primary and secondary outcomes according to isolated pathogens and infective syndrome

	Total number	Complete resolution	Partial resolution	No resolution	Not enough data
Causative pathogen, n (%)					
- Gram positive bacteria	28 (34.6)	7 (25)*	17 (60.7)	0 (0)	4 (14.3)
- Gram negative bacteria	53 (65.4)	21 (39.6)*	28 (52.8)	1 (100)	3 (5.7)
Infective Syndrome, n (%)					
- Bloodstream infections	11 (19.6)	4 (36.4)^	4 (36.4)	0 (0)	3 (27.3)
- Respiratory tract infections	23 (41.1)	4 (17.4)^	17 (73.9)	1 (4.3)	1 (4.3)
- Urinary tract infections	9 (16.1)	7 (77.7)^	2 (22.2)	0 (0)	0 (0)
- Bone Infections	9 (16.1)	4 (44.4)^	5 (55.5)	0 (0)	0 (0)
- Surgical site infections	4 (7.1)	3 (75)^	0 (0)	0 (0)	0 (0)

*p=0.32 for complete resolution vs partial or no resolution according to type of causative pathogen

^p=0.007 for complete resolution vs partial or no resolution according to infective syndrome

Supplementary Table S5. Univariate analysis of factors associated with electrolyte imbalance development during fosfomycin administration.

	Electrolyte Imbalance	No Electrolyte Imbalance	*p-value*
Number of patients, n (%)	16 (29.6)	38 (67.8)	
CKD, n (%)	7 (43.7)	5 (13.1)	*0.02*
Heart Failure, n (%)	2 (12.5)	4 (10.5)	*0.58*
Potassium Sparing Diuretics, n (%)	2 (12.5)	2 (5.2)	*0.57*
Saline Solution Dilution, n (%)	2 (12.5)	9 (23.7)	*0.47*
Glucose Solution Dilution, n (%)	10 (17.8)	23 (60.5)	*0.50*
Basal Sodium, median [IQR]*	139 [138-140]	137 [134-140]	*0.77*
Basal Potassium, median [IQR]°	4.0 [3.6-5.0]	4.1 [3.7-4.6]	*0.09*

p-value was generated by ANOVA test and Pearson's chi-square test for median and frequency values, respectively. IQR: Inter Quartile Range

**Sodium imbalance defined as sodium levels > 145 (upper limit of normal)*

°Potassium imbalance defined as potassium levels < 3.5 (lower limit of normal)
